# Malaria prevalence in Mangaluru city area in the southwestern coastal region of India

**DOI:** 10.1186/s12936-017-2141-0

**Published:** 2017-12-19

**Authors:** Kiran K. Dayanand, Kishore Punnath, Valleesha Chandrashekar, Rajeshwara N. Achur, Srinivas B. Kakkilaya, Susanta K. Ghosh, Suchetha Kumari, D. Channe Gowda

**Affiliations:** 1 Department of Biochemistry and Molecular Biology, The Pennsylvania State University College of Medicine, 500 University Drive, Hershey, PA 17033 USA; 2 Department of Biochemistry, K. S. Hegde Medical Academy, NITTE University, Mangaluru, India; 3 Department of Biochemistry, Kuvempu University, Shankaraghatta, Shivamogga District, Karnataka India; 4 Light House Polyclinic, Light House Hill Road, Mangaluru, Karnataka India; 5 Department of Biological Control, National Institute of Malaria Research, Poojanahalli, Bangalore, India

**Keywords:** Mangaluru city, India, Malaria, *Plasmodium vivax* and *P. falciparum*, Prevalence, Socio-demographic factors

## Abstract

**Background:**

Malaria is highly prevalent in many parts of India and the Indian subcontinent. Mangaluru, a city in the southwest coastal region of Karnataka state in India, and surrounding areas are malaria endemic with 10–12 annual parasite index. Despite high endemicity, to-date, very little has been reported on the epidemiology and burden of malaria in this area.

**Methods:**

A cross-sectional surveillance of malaria cases was performed among 900 febrile symptomatic native people (long-time residents) and immigrant labourers (temporary residents) living in Mangaluru city area. During each of dry, rainy, and end of rainy season, blood samples from a group of 300 randomly selected symptomatic people were screened for malaria infection. Data on socio-demographic, literacy, knowledge of malaria, and treatment-seeking behaviour were collected to understand the socio-demographic contributions to malaria menace in this region.

**Results:**

Malaria is prevalent in Mangaluru region throughout the year and *Plasmodium vivax* is predominant species compared to *Plasmodium falciparum*. The infection frequency was found to be high during rainy season. Infections were markedly higher in males than females, and in adults aged 16–45 years than both younger and older age groups. Also, malaria incidence was high among immigrants compared to native population. In both groups, infection rate was directly correlated with their literacy level, knowledge on malaria, dwelling environment, and protective measures used. There was also a significant difference in treatment-seeking behaviour between these two groups.

**Conclusions:**

Malaria incidences in Mangaluru region are predominantly localized to certain hotspot areas within the city, where socioeconomically underprivileged and immigrant labourers are densely populated. These areas have inadequate sanitation and constant water stagnation, harbouring high vector density and contributing to high infection incidences. Additionally, people in these areas seldom practice preventive measures such as using bed nets. The high incidences of malaria in adults are due to minimal cloth wearing, and long working hours stretching to late evenings in places with high vector density. Instituting heightened preventive public measures by governments and creating awareness on using preventive protective and environmental hygienic measures through educational programmes may substantially reduce the risk of contracting infections in these areas and spreading to other areas.

## Background

Malaria is one of the most prevalent parasitic diseases worldwide, currently accounting for an estimated 200–300 million clinical cases every year and about half a million deaths worldwide [1]. Approximately 50% of the world’s population lives in malaria endemic regions and the disease is a significant contributor to the social and economic progress of people in many developing countries, particularly in sub-Saharan Africa, Southeast Asia, and South America [2, 3]. Although majority of malarial deaths are in Africa, substantial number of deaths also occur in the Southeast Asia and South America. India accounts for about 70% of total malaria cases in Southeast Asia region followed by Indonesia and Myanmar [4, 5]. In India, about 82% of the population is at risk of malaria with an estimated 1.5 million clinical cases annually [6].

Malaria is highly endemic and persistent throughout the year in several parts of southwestern regions of India, including a substantial portion of Karnataka state [7, 8]. Mangaluru is the government headquarters of Dakshina Kannada district in Karnataka state. Malaria is endemic in Dakshina Kannada district, which receives high rainfall during rainy season and exhibits humid tropical environment, harbouring high vector density and contributing to high incidences of malaria. In the last two decades, increase in building and road construction activities as a part of rapid urbanization resulted in a substantial number of immigrant labourers from other parts of India, prominently from Northeastern regions, where malaria is highly endemic, migrating to Mangaluru city. This resulted in the spread and high incidences of malaria in Mangaluru city and surrounding areas [8].

Malaria persists in Mangaluru area throughout the year with peak infections in the rainy season. Several localities in the city and suburban areas harbor slum dwellers and socio-economically disadvantaged people. Because of this and wide-spread stagnant water, these localities are hotspots for malaria infection throughout the year with high incidence rates in the rainy season [9]. Despite high endemicity and huge health burden, to-date very little has been reported on malaria prevalence and on socioeconomic and demographic factors that contribute to malaria incidences in Mangaluru region. Government records, which are mainly based on the number of symptomatic patients diagnosed at major hospitals in Mangaluru city, indicate that around 6000–7000 malaria cases occur annually. These numbers could be underestimates since asymptomatic cases, and those that receive treatment in some hospitals and private clinics are not included in the records. Given this scenario, the main objective of this study was to determine the extent of malaria prevalence and assess the demographic factors that contribute to malaria hotspots in Mangaluru city.

## Methods

### Study site

**Fig. 1:**
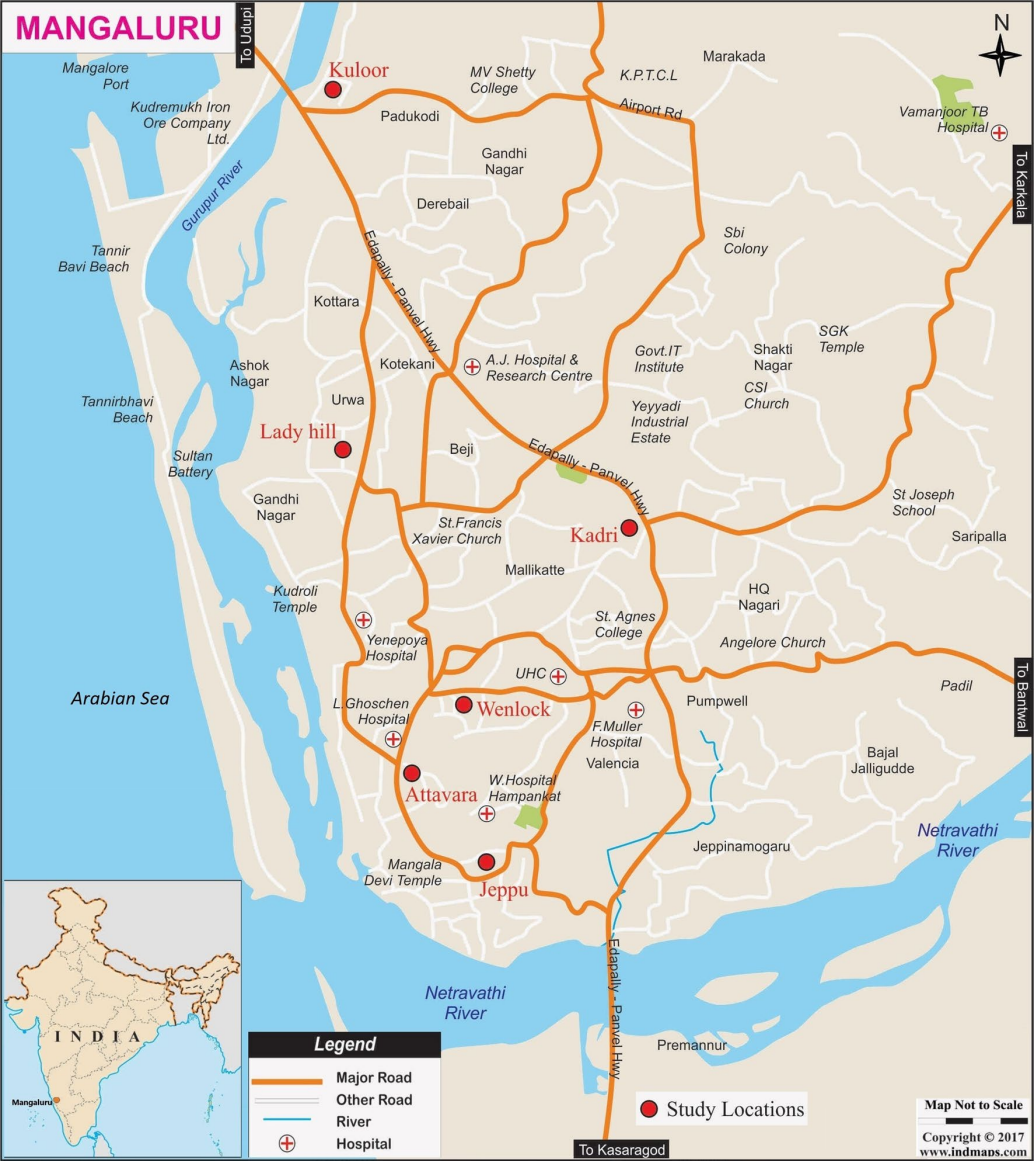
Mangaluru city map showing the malaria hotspots in the city

Mangaluru city is situated 12.91°N, 74.85°E on the basin of rivers Netravathi and Gurupura in the Arabian peninsula of Dakshina Kannada district (Fig. 1). The city and its surrounding areas have a tropical climate, with high rainfall and temperature varying from 17 °C at night to 38 °C during day times. The warm and humid climate of Mangaluru city and its surrounding areas provide an ideal environment for the breeding of mosquitoes and disease transmission. Thus, this area harbours high vector density and has high incidences of malaria.

### Study design and population

A community-based cross-sectional study was conducted among febrile-symptomatic individuals residing in malaria hotspot areas in Mangaluru city during February to December 2014. Passive case detection was performed at three different time points in the year, February–March (dry season), August–September (rainy reason), and November–December (the end of rainy season). Multi stage random sampling technique was used in recruiting individuals. A one-sample study design within a population of 5,00,000 city residents was used to determine the sample size, assuming that the malaria risk prevalence is between 20 and 30% of the city population. The study was focused on selected malaria hotspots, where socioeconomically disadvantaged and immigrant workers live. In each season, 300 individuals were randomly recruited based on household sampling frame. Altogether, 900 individuals were screened during the calendar year of 2014. Individuals diagnosed to have malaria infection and did not receive treatment were referred to nearby government hospitals or health care facilities for treatment.

### Malaria diagnosis

Symptomatic individuals were screened for malaria infection using Giemsa-stained thin and thick blood film examination, and by using a malaria Ag Pf/Pv rapid diagnostic test kit (SD Bioline, India), which detects histidine-rich protein II antigen of *Plasmodium falciparum* and lactate dehydrogenase of *Plasmodium vivax* in human blood. Individuals who were positive for infection and those who have already been diagnosed in hospitals and clinics, and those were undergoing treatment at the time of study were recruited.

### Study tool

A semi-structured interview questionnaire was used to collect information of the study population that included socio-demographic factors, resident status (whether they are long-time residents or migrated recently to this region for the livelihood), recent travel history, and education level. Information about knowledge on the disease, how it spreads and transmits, preventive measures, and where to get diagnosed and obtain proper treatment was collected. Other information collected was treatment-seeking behaviours, such as measures taken after being sick, symptoms experienced during sickness, and previous history of malaria, if any.

### Statistical analysis

Statistical analysis of data was performed using Statistical Package for the Social Sciences (SPSS) for Windows, Version 23.0. Chicago, SPSS Inc. Statistical significance was derived using Chi square test and by logistic regression.

## Results

### The study group characteristics

The study was conducted during February to December 2014 at six different localities, where low-income group dwellers in Mangaluru city corporation limits reside. These localities are considered as malaria hotspots in the city (Fig. 1). Of the total 900 febrile symptomatic individuals screened for malaria infection at three time points (300 subjects at each time point) in the year—dry season (February–March), rainy season (August–September), and end of rainy season (November–December), 451 (50.1%) were native individuals and 449 (49.9%) were non-native immigrants, and 273 (30.3%) were females and 627 (69.7%) males (Table 1). The study population was stratified into different age groups in which, 61 (6.8%) were 2–15 years old, 324 (36.0%) were 16–30 years old, 330 (36.7%) 31–45 years old, 150 (16.7%) 46–60 years old, and 35 (3.9%) 61–75 years old. With respect to levels of education, 337 (37.4%) were literates but had no formal education, 343 (38.1%) had primary education, 205 (22.8%) had secondary education, and 15 (1.7%) had college education. Based on profession, 439 (48.8%) were building or road construction workers, 42 (4.7%) were hotel workers, 76 (8.4%) were porters, 62 (6.9%) were school students, and 281 (31.2%) were homemakers as well as those belonging to other occupations (see Table 1).

**Table 1:** Socio-demographic characteristics of study participants

	Immigrants		Native		Total	
	n	%	n	%	n	%
Gender						
Female	89	19.8	184	40.8	273	30.3
Male	360	80.2	267	59.2	627	69.7
Total	449	100	451	100	900	100
Age						
2–15	10	2.2	51	11.3	61	6.8
16–30	150	33.4	174	38.6	324	36.0
31–45	204	45.4	126	27.9	330	36.7
46–60	74	16.5	76	16.9	150	16.7
61–75	11	2.4	24	5.3	35	3.9
Total	449	100	451	100	900	100
Education						
Uneducated	191	42.5	146	32.4	337	37.4
Primary	181	40.3	162	35.9	343	38.1
Secondary	75	16.7	130	28.8	205	22.8
College education	2	0.4	13	2.9	15	1.7
Total	449	100	451	100	900	100
Occupation						
Construction worker	337	75.1	102	22.6	439	48.8
Hotel worker	17	3.8	25	5.5	42	4.7
Porter	36	8.0	40	8.9	76	8.4
Student	8	1.8	54	12.0	62	6.9
Others	51	11.4	230	51	281	31.2
Total	449	100	451	100	900	100
Screening time period						
Feb–March	150	33.4	150	33.3	300	33.3
Aug–Sep	149	33.2	151	33.5	300	33.3
Nov–Dec	150	33.4	150	33.3	300	33.3
Total	449	100	451	100	900	100
Previous history of malaria						
Yes	181	40.4	228	50.8	409	45.6
No	267	59.6	221	49.2	488	54.4
Unsure	1	0.2	2	0.4	3	0.3
Total	449	100	451	100	900	100

### Parasite species and infection prevalence in native and immigrant people

Of the total 900 febrile symptomatic individuals people screened, 449 had detectable malaria infections and the remainder had no detectable parasite by microscopic examination (Table 2). Regardless of gender, age, and residence status (native or immigrants), *P. vivax* infections were more prevalent than *P. falciparum* infections. Of the total 900 people, regardless of residence status, 367 (81.7%), 67 (14.9%) and 15 (3.3%), respectively, had *P. vivax*, *P. falciparum*, and *P. vivax* and *P. falciparum* mixed infections (Table 2). The distribution of *P. vivax*, *P. falciparum*, and *P. vivax* and *P. falciparum* mixed infection prevalence was similar among males and females, i.e., ~ 80, ~ 17 and ~ 3%, respectively. Irrespective of residence status, infection was highest among males 329 (73.3%) than females 120 (26.7%). Infection in immigrant labourers 272 (60.5%) was higher compared to native population 177 (39.4%) and in both groups, *P. vivax* was the predominant infection (P = 0.001). Ratio of male to female among infected immigrants was ~ 4:1 as compared to 2:1 in native group. With regard to occupational status, highest prevalence of infection 250 (55.7%) was seen among construction workers compared to 17 (3.8%) in hotel workers, 40 (8.9%) in porters, 24 (5.3%) in students, and 118 (26.3%) in all other occupations (Table 2). In both native and immigrant groups, infection was predominantly seen in adults; 157 (35%) were 16–30 years old, 172 (38.3%) were 31–45 years old, 26 (5.8%) were 2–15 years old, 82 (18.3%) were 46–60 years old, and 12 (2.7%) were 61–75 years old (Table 2). Regarding educational status, 179 (39.9%) did not have any formal education, 167 (37.2%) had primary, 94 (20.9%) had secondary education, and 9 (2.0%) had college education.

**Table 2:** Logistic regression analysis of demographic factors associated with infection

	Infection				χ^2^	P value	Adjusted odds ratio (95% CI)
	Present		Absent				
	n	%	n	%			
Gender							
Female	120	26.7	153	33.9	5.52	0.019	1.40 (1.05–1.87)
Male	329	73.3	298	66.1			
Age (years)							
2–15	26	5.8	35	7.8	6.99	0.136	NS
16–30	157	35.0	167	37.0			
31–45	172	38.3	158	35.0			
46–60	82	18.3	68	15.1			
61–75	12	2.7	23	5.1			
Infecting parasite species							
*P. vivax*	367	81.7	0				
*P. falciparum*	67	14.9	0				
Mixed (Pv and Pf)	15	3.4	0				
Education							
Uneducated	179	39.9	158	35.0	3.55	0.314	NS
Primary	167	37.2	176	39.0			
Secondary	94	20.9	111	24.6			
College education	9	2.0	6	1.3			
Occupation							
Construction worker	250	55.7	189	41.9	20.57	0.008	2.09 (1.21–3.61)
Hotel worker	17	3.8	25	5.5		0.856	1.07 (0.48–2.39)
Porter	40	8.9	36	8.0		0.104	1.7 (0.89–3.47)
Student	24	5.3	38	8.4			
Others	118	26.3	163	36		0.635	1.14 (0.65–2.01)

*NS* not significant

### Infection and parasite prevalence during different seasons

Malaria was persistent throughout the year in Mangaluru. Among 449 infected people in the study group, infections were higher during the middle of the rainy season (August–September) and at the end of rainy season (November–December), respectively, 179 (59.6%) and 146 (48.7%) as compared to 124 (41.3%) during the dry season (February–March) (Table 3). The main symptoms among the study participants in general were fever, headache, chills, vomiting, and nausea. Symptoms exhibited specifically by *P. falciparum*, and *P. vivax*-infected individuals, and those who had mixed infections are given in Table 4. Of the total 272 infected immigrants in the study group, 111 (45.8%) individuals had one or more episodes of malaria in their lifetime. Among 177 native infected-people in the study group, 90 (50.8%) had previous malaria infections.

**Table 3:** Seasonal variations in malaria infection

Time point of screening	Infection				χ^2^	P value	Adjusted odds ratio (95% CI)
	Present		Absent				
	n	%	n	%			
Feb–March	124	41.3	176	58.6	20.43		
Aug–Sep	179	59.6	121	40.3		0.001	2.10 (1.51–2.90)
Nov–Dec	146	48.7	154	51.3		0.071	1.34 (0.97–1.85)

The P values for the data during Aug–Sep and Nov–Dec are in comparison to those during Feb–Mar

**Table 4:** Symptoms in malaria infected and non-infected febrile individuals

	Type of infection					
	Pv		Pf		Mixed	
	Count	%	Count	%	Count	%
Symptoms						
Absent	0	0	0	0	0	0
Present	367	100	67	100	15	100
Total	367	100	67	100	15	100
Fever						
Absent	55	14.5	13	19.40	6	40
Present	312	85.5	54	80.60	9	60
Total	367	100	67	100	15	100
Headache						
Absent	231	62.7	45	67.2	11	73.30
Present	136	37.3	22	32.8	4	26.7
Total	367	100	67	100.0	15	100
Vomiting						
Absent	366	99.7	66	98.5	15	100
Present	1	0.3	1	1.5	0	0
Total	367	100	67	100	15	100
Nausea						
Absent	22	5.5	8	11.9	0	0
Present	345	94.5	59	88.1	15	100
Total	367	100	67	100	15	100

### Association of malaria prevalence with socio-economic and education status as well as malaria knowledge among native and non-native population

In the immigrant’s study group, construction workers were at high risk of infection; of the total 272 infected immigrants, 211 (77.6%) were construction workers as compared to 10 (3.7%) hotel workers, 2 (0.7%) students, 22 (8.1%) porters, and 27 (9.9%) other occupations (Table 5). On the other hand, among native study group population, infections among constructions workers was relatively low, which is 39 (22%) of 177 total individuals, and the remainder were 7 (4%) hotel workers, 22 (12.4%) students, 18 (10.2%) porters, and 91 (51.4%) were with other occupations (Table 5). The majority of individuals in infected migrant study group, 226 (83%) of total 272, was either uneducated or had only basic primary education. In the case of infected native people, 120 (67.7%) were either uneducated or had only basic primary education (Table 5). The majority (98.3%) of the native study population was knowledgeable about malaria infection, its mode of transmission and preventive measures, whereas in the immigrant group 69% had this information.

**Table 5:** Demographic information of native and immigrant respondents

	Study participants					
	Immigrants		Native		Total	
	n	%	n	%	n	%
Gender						
Male	217	79.8	112	63.3	329	73.3
Female	55	20.2	65	36.7	120	26.7
Age						
2–15	6	2.2	20	11.3	26	5.80
16–30	86	31.6	71	40.1	157	35
31–45	122	44.9	50	28.2	172	38.3
46–60	51	18.8	31	17.5	82	18.3
61–75	7	2.6	5	2.8	12	2.7
Education						
No formal education	126	46.3	53	29.9	179	39.9
Primary	100	36.8	67	37.9	167	37.2
Secondary	44	16.2	50	28.2	94	20.9
Degree	2	0.7	7	4.0	9	2.0
Occupation						
Construction worker	211	77.6	39	22.0	250	55.7
Hotel worker	10	3.7	7	4.0	17	3.8
Porter	22	8.1	18	10.2	40	8.9
Student	2	0.7	22	12.4	24	5.3
Others	27	9.9	91	51.4	118	26.3
Screening time period						
Feb–March	78	28.7	46	26.0	124	27.6
Aug–Sep	106	39.0	73	41.2	179	39.9
Nov–Dec	88	32.4	58	32.8	146	32.5
Previous history of malaria						
Yes	111	41.0	90	50.8	201	44.80
No	160	59.0	87	49.2	247	55

Treatment-seeking behaviour of all the 900 study participants was recorded. In both native and immigrant groups, the preferred treatment was anti-malarial drugs. In this region, chloroquine is the prescribed drug of treatment for uncomplicated *P. vivax* infection and artemisinin-based combination therapy comprising artesunate, sulfadoxine and pyrimethamine is prescribed for uncomplicated *P. falciparum* infection. Primaquine is prescribed as a radical treatment for both the types of infections [10]. Among immigrants, out of total 272 infected individuals, 75 (27.5%) went to clinics for treatment after feeling sick, 107 (39.3%) took medication from the local pharmacy stores after feeling sick instead of going to clinic for treatment, and the majority of them were given antipyretics and analgesics for symptomatic treatment by the drug stores pharmacist (Table 6). Only 2 (0.7%) preferred home remedy and 88 (32.3%) did not take any treatment after feeling sick. In case of native people, out of total 177 infected individuals, 82 (46.3%) visited clinics/hospitals, 59 (33.3%) took medication from local pharmacy, 4 (2.2%) preferred home remedy, and 32 (18%) did not take any treatment after feeling sick.

**Table 6:** Treatment seeking behavior among malaria infected native and immigrant population

	Group				P value
	Immigrants		Native		
	n	%	n	%	
Measures taken after feeling sick					
Allopathy clinic	75	27.5	82	46.3	0.001
Herbal medication	0	0	1	0.5	
Drug seller	107	39.3	59	33.3	
Home remedy	2	0.7	4	2.2	
No measure was taken	88	32.3	32	18	
Treatment time after feeling sick					
Within 2–4 days	128	47.0	94	53.1	0.059
No delay within 24 h	60	22.0	47	26.5	
No treatment taken	84	30.8	37	20.9	
Reason for delay in treatment					
Not aware where to go	42	15.4	14	7.9	0.076
Self-medication	19	6.9	18	10.1	
Financial issues	80	29.4	42	23.7	

Regarding treatment-seeking, of 177 total infected native individuals, 94 (53.1%) waited 2–4 days for taking treatment after feeling sick, about 47 (26.5%) went on the same day for medication, and 37 (20.9%) did not take any treatment (Table 6). In the case of non-native individuals, 128 (47%) of total 272 waited for 2–4 days to go to clinics/hospitals for taking treatment after feeling sick, 60 (22%) went on the same day to clinic for medication, and 84 (30.8%) had not taken any treatment. The main reason for delay in seeking treatment among natives was that 18 (10.1%) had tried self-medication i.e., home remedy for the treatment, 42 (23.7%) had financial reasons, and 14 (7.9%) were not aware as to where to go for the diagnosis of malaria and 10 (6.2%) had other reasons such as having no time, clinics/hospitals being too far away, or lethargic to take medication; in the remaining 93 (52.5%) individuals there was no delay in treatment. In the case of immigrants, 19 (6.9%) had tried self-medication, 80 (29.4%) had financial limitation, and 42 (15.4%) were not aware where to go for the diagnosis, and 18 (6.6%) had other reasons, for example, not finding time to seek treatment. In remaining 113 (41.5%) individuals, there was no delay in treatment (Table 6). Based on knowledge of how malaria spreads, 307 (68%) of 451 total native individuals replied that the infection spreads through the bite of mosquitoes, 47 (10.4%) said infection was due to lack of cleanliness, 38 (8.4%) said due to fly/insect bite, and some gave multiple answers (Table 7). Among immigrants, 232 (51.6%) of 449 thought malaria spreads through the bite of mosquito, 38 (8.4%) said due to lack of cleanliness, and 41 (9.1%) said due to fly/insect bite. Regarding how malaria can be prevented, 240 (53.2%) of 451 native individuals said by using bed nets while sleeping, 28 (6.2%) said by using mosquito repellents, and 109 (24.1%) said by limiting the breeding sources. Among immigrant group, 140 (31.1%) of 449 said by using bed nets while sleeping, 23 (5.1%) said by using mosquito repellents, 60 (13.3%) said by limiting the breeding sources (Table 7). With respect to knowledge on risk of infection due to stagnant water storage, not using bed nets, travelling to endemic places, and sleeping in those places with high vector density, was significantly higher in native individuals compared to non-native individuals.

**Table 7:** Knowledge on malaria among native and immigrant population

	Group				P value
	Immigrants		Native		
	n	%	n	%	
How does a person get malaria					
Mosquito bite	232	51.6	307	68	0.333
Fly/insect bite	41	9.1	38	8.4	
Lack of cleanliness	38	8.4	47	10.4	
How malaria can be prevented					
Eliminating breeding sources	60	13.3	109	24.1	0.451
Bed nets	140	31.1	240	53.2	
Mosquito repellents	23	5.1	28	6.2	

## Discussion

The aim of this study was to understand the burden of malaria in Mangaluru city area. For the past several decades, malaria is endemic in this city and its surrounding areas with peak infections occurring during rainy season. Although *P. vivax* and *P. falciparum* are prevalent throughout the year, the former predominates. The prevalence of *P. vivax*, *P. falciparum* and mixed infections was, respectively, 80, ~ 17 and ~ 3%. These results are consistent with the data recorded in Mangaluru city area by the District Vector Borne Disease Control Programne (DVBDCP) office of Dakshina Kannada District, which shows that ~ 80% *P. vivax* and ~ 20% *P. falciparum* infections. The data recorded by DVBDCP indicate that Mangaluru city area has been having a similar ratios of *P. falciparum* and mixed infections since 1990 [7, 8]. As per the report published by National Vector Borne Disease Control Programme, the average *P. falciparum* and *P. vivax* infection throughout India is in the ratio of 1.5:1. However the proportion of *P. vivax* and *P. falciparum* infection varies in different parts of India. The prevalence of *P. vivax* and *P. falciparum* infections in Indo-Gangetic plains and Northern hilly states, Northwestern and Southwestern regions is 80–90% *P. vivax* and 10–20% *P. falciparum* respectively; however, in the forest areas of South Eastern regions inhabited by ethnic tribes, the situation is markedly different with *P. vivax* to *P. falciparum* prevalence ratio of 1:3 [6, 11, 12].

In Mangaluru, the incidence of malaria infection is highest among immigrant workers compared to native individuals. This is due to a rapid urbanization and construction activity, which provides many employment opportunities for immigrant workers. So, labourers mainly from Northern and Northeastern states of India, where malaria is endemic, come to Mangaluru. They reside in temporary accommodations, often in the construction sites with poor living conditions, disposing to malaria infection. Water stored at the construction sites and stagnant water in building under constructions provides ideal breeding conditions for the mosquito and spreading of the disease. Additionally, limited or lack of health knowledge on malaria contributes to accelerated transmission of infection. Individuals who travel to malaria endemic places in other parts of Karnataka and India also are the source of spreading malaria in Mangaluru. Especially, the migrant workers from Northern or Northeastern parts of India where malaria is highly endemic are high carriers of infection, contributing markedly to spread of malaria locally [13–16]. Thus it appears that travelling of immigrants back and forth to their native places contributes to perpetual spreading of malaria in Mangaluru, despite control measures in place.

Unlike in Africa and several others parts of endemic regions in the world, where infection is predominantly in the age group of < 12 years [17, 18], the majority of infection in Mangaluru area is in adult age groups, 16–45 years old. The latter is also the case in other parts of India and other South Asian countries [19]. The results presented here also show that the majority of infections were in adult construction workers, particularly in immigrants. A similar trend was also seen in a recent study from this region [20]. This is because construction workers were mainly immigrant workers in the age group of 16–45 years. These people work until late evenings, during the time period at which the mosquito-feeding activity is high. In addition, these workers wear minimum clothes because of humid weather and hard physical labor, exposing to high risk of mosquito bites. Moreover, these people reside in temporary accommodations, where mosquito prevalence is high, and they do not use preventive measures such as bed nets while sleeping during nights. Since the majority of constructions workers are males, infections are more prevalent in males compared to females. These are the reasons why malaria is also highly prevalent in utensil-cleaning workers at hotels and hostels. Thus, malaria in Mangaluru city area is mainly occupational-related illness.

As in the case of other malaria endemic countries or other parts of India [21, 22], the majority of native people in the current study area are aware that malaria is transmitted by the bite of mosquito. However, a significant number of people in low socioeconomic and semiliterate group and migrant population had no knowledge about how malaria spreads. Knowledge on the use of bed nets as a preventive measure against mosquito bite and its usage was high among the native people, explaining relatively low incidences of infections among this population.

Although the majority of study group preferred the prescribed drug treatment, a significant number of both native and migrant groups preferred self-medication, such as steam inhalation for nasal congestions, and herbal preparation for fever during initial period of experiencing symptoms. In the migrant population, who sought drug treatment, the majority went to the informal medicine providers, such as drug stores and ended up in buying antipyretics, analgesics, antiemetic and antihistamines. Thus, educating vulnerable people on malaria knowledge and on implementing preventative measures, and the necessity of seeking early diagnosis and prompt treatment may prove to be effective in controlling malaria in Mangaluru area.

## Conclusions

Malaria is highly prevalent in Mangaluru and persists throughout the year with maximum level of infection cases seen during the rainy season. Both *P. vivax* and *P. falciparum* infections occur throughout the year at ~ 80 and ~ 20% proportions, respectively. Overall, the infection rate is significantly higher among immigrant and socioeconomically disadvantaged temporary resident laborers, who work for house and building construction industries, hotels, and hostels, where stagnant water harbours high vector density. In both native and immigrant classes of people, males were highly disposed to the infection due to their long working hours that include late evening when feeding activity of vector is high. Also, due to hot and humid weather condition, the workers wear minimal clothing. Unsatisfactory levels of malaria knowledge and lack of preventive protective measures are substantial risk factors in immigrant population. Efforts in seeking treatment and knowledge about malaria infection are low among immigrant population compared to native people. Immigrant individuals are also largely unaware of the government facilities that provide free diagnosis and treatment for malaria. Their lack of knowledge appears to be mainly attributed to limited levels of health education activities in malaria hotspot localities in the city. Therefore, conducting educational programmes on malaria awareness and implementation of public control measures are likely to significantly reduce infection rates in Mangaluru.
